# A bulk segregant transcriptome analysis reveals metabolic and cellular processes associated with *Orange* allelic variation and fruit β-carotene accumulation in melon fruit

**DOI:** 10.1186/s12870-015-0661-8

**Published:** 2015-11-09

**Authors:** Noam Chayut, Hui Yuan, Shachar Ohali, Ayala Meir, Yelena Yeselson, Vitaly Portnoy, Yi Zheng, Zhangjun Fei, Efraim Lewinsohn, Nurit Katzir, Arthur A. Schaffer, Shimon Gepstein, Joseph Burger, Li Li, Yaakov Tadmor

**Affiliations:** Plant Science Institute, Agricultural Research Organization, Newe Ya’ar Research Center, P.O. Box 1021, Ramat Yishay, 30095 Israel; Faculty of Biology, Technion – Israel Institute of Technology, Haifa, 32000 Israel; Plant Breeding and Genetics Section, School of Integrative Plant Science, Cornell University, Ithaca, NY 14853 USA; Boyce Thompson Institute for Plant Research, Cornell University, Ithaca, NY 14853 USA; Plant Science Institute, Agricultural Research Organization, The Volcani Center, P.O.B. 6, Bet-Dagan, 50250 ISRAEL; US Department of Agriculture–Agricultural Research Service, Robert W Holly Center for Agriculture and Health, Cornell University, Ithaca, NY 14853 USA

**Keywords:** Melon, *Cucumis melo*, Carotenoids, Beta-carotene, Bulk segregant analysis, *CmOr*, Fruit development, Transcriptome

## Abstract

**Background:**

Melon fruit flesh color is primarily controlled by the “golden” single nucleotide polymorhism of the *“Orange”* gene, *CmOr*, which dominantly triggers the accumulation of the pro-vitamin A molecule, β-carotene, in the fruit mesocarp. The mechanism by which *CmOr* operates is not fully understood. To identify cellular and metabolic processes associated with *CmOr* allelic variation, we compared the transcriptome of bulks of developing fruit of homozygous orange and green fruited F_3_ families derived from a cross between orange and green fruited parental lines.

**Results:**

Pooling together F_3_ families that share same fruit flesh color and thus the same *CmOr* allelic variation, normalized traits unrelated to *CmOr* allelic variation. RNA sequencing analysis of these bulks enabled the identification of differentially expressed genes. These genes were clustered into functional groups. The relatively enriched functional groups were those involved in photosynthesis, RNA and protein regulation, and response to stress.

**Conclusions:**

The differentially expressed genes and the enriched processes identified here by bulk segregant RNA sequencing analysis are likely part of the regulatory network of *CmOr*. Our study demonstrates the resolution power of bulk segregant RNA sequencing in identifying genes related to commercially important traits and provides a useful tool for better understanding the mode of action of *CmOr* gene in the mediation of carotenoid accumulation.

**Electronic supplementary material:**

The online version of this article (doi:10.1186/s12870-015-0661-8) contains supplementary material, which is available to authorized users.

## Background

**Fig. 1:**
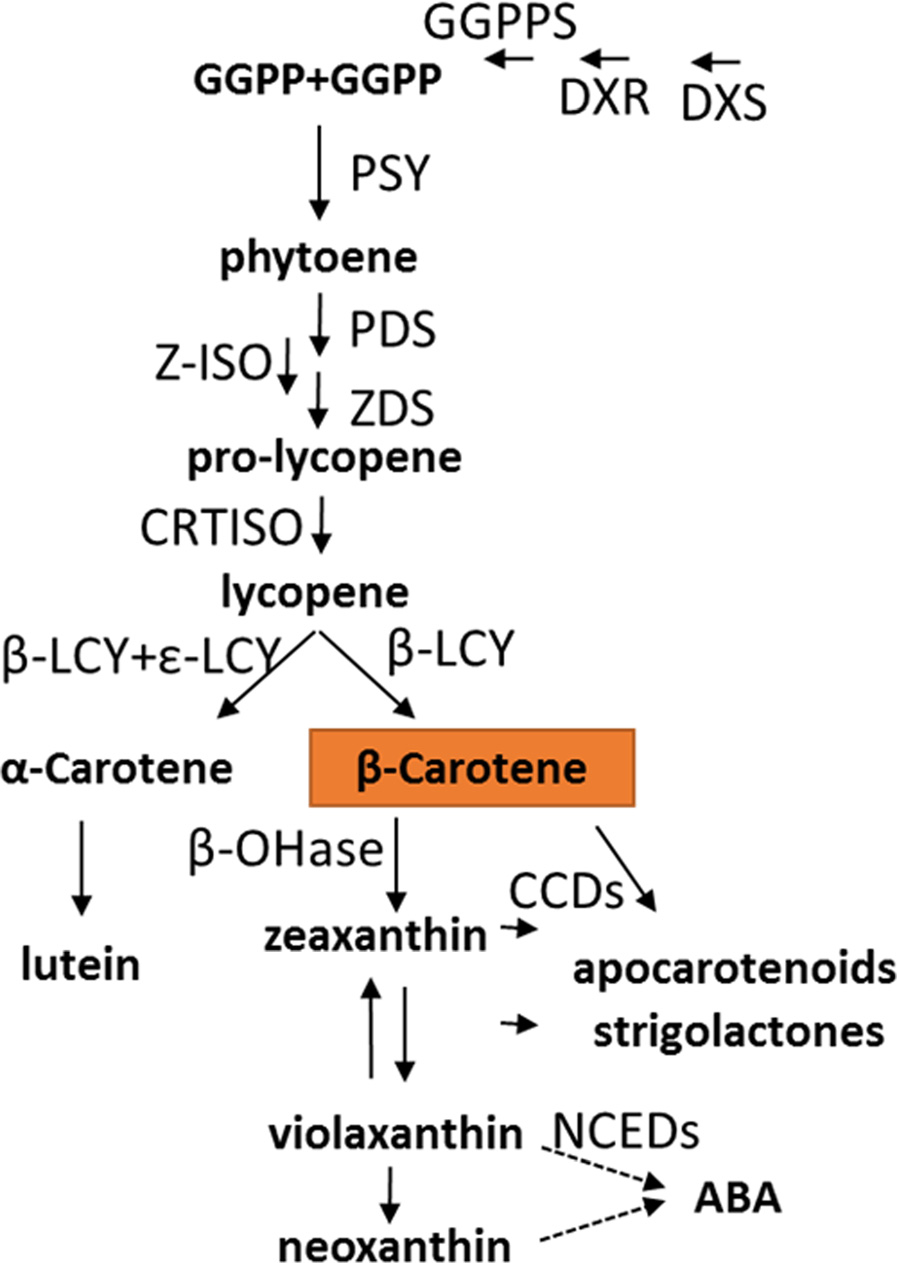
Schematic presentation of the metabolic pathway leading to β-carotene formation and major downstream products. Enzymes are aligned with arrows. The three upstream enzymes belonging to the methylerthritol-4-phosphate (MEP) pathway are: deoxy-d-xylulose 5-phosphate (DXP) synthase (DXS), DXP reductoisomerase (DXR) and geranylgeranyl diphosphate synthase (GGPPS). The carotenogenesis enzymes are: phytoene synthase (PSY); phytoene desaturase (PDS); ζ-carotene isomerase (Z-ISO); ζ-carotene desaturase (ZDS); carotene isomerase (CRTISO). lycopene ɛ-cyclase (ε-LCY); lycopene β-cyclase (β-LCY); β-carotene hydroxylase (β-OHase) carotenoid cleavage dioxygenases (CCDs); 9-cis-epoxycarotenoid dioxygenases (NCEDs); Enzyme names and abbreviations are after [1]

Carotenoids in fruits have been subjected to extensive studies due to their nutritional and visual appealing importance. The metabolic pathway leading to the accumulation of carotenoids in plants has been well elucidated and extensively reviewed by many authors, including recently by Nisar et al. [1]. A scheme of the carotenoid biosynthetic pathway is illustrated in Fig. 1. Some carotenoids such as β-carotene serve as precursors of vitamin A, some are potent antioxidants and many carotenoids are believed to provide protection against certain cancers, heart diseases, and age-related eye disease [2–4]. A large number of fruits owe their vivid color to carotenoid accumulation. In fleshy fruits, carotenoid level and composition vary dramatically among species and within different varieties of the same species. Because carotenoids confer fruit color, their evolutionary role in fruit is likely to attract seed dispersers. Carotenoids also constitute an important economical trait in horticulture. In addition, carotenoid breakdown products have profound effects in fruit flavor and aroma, which may have further attractive effects on seed dispersers and consumers [5–10].

**Fig. 2:**
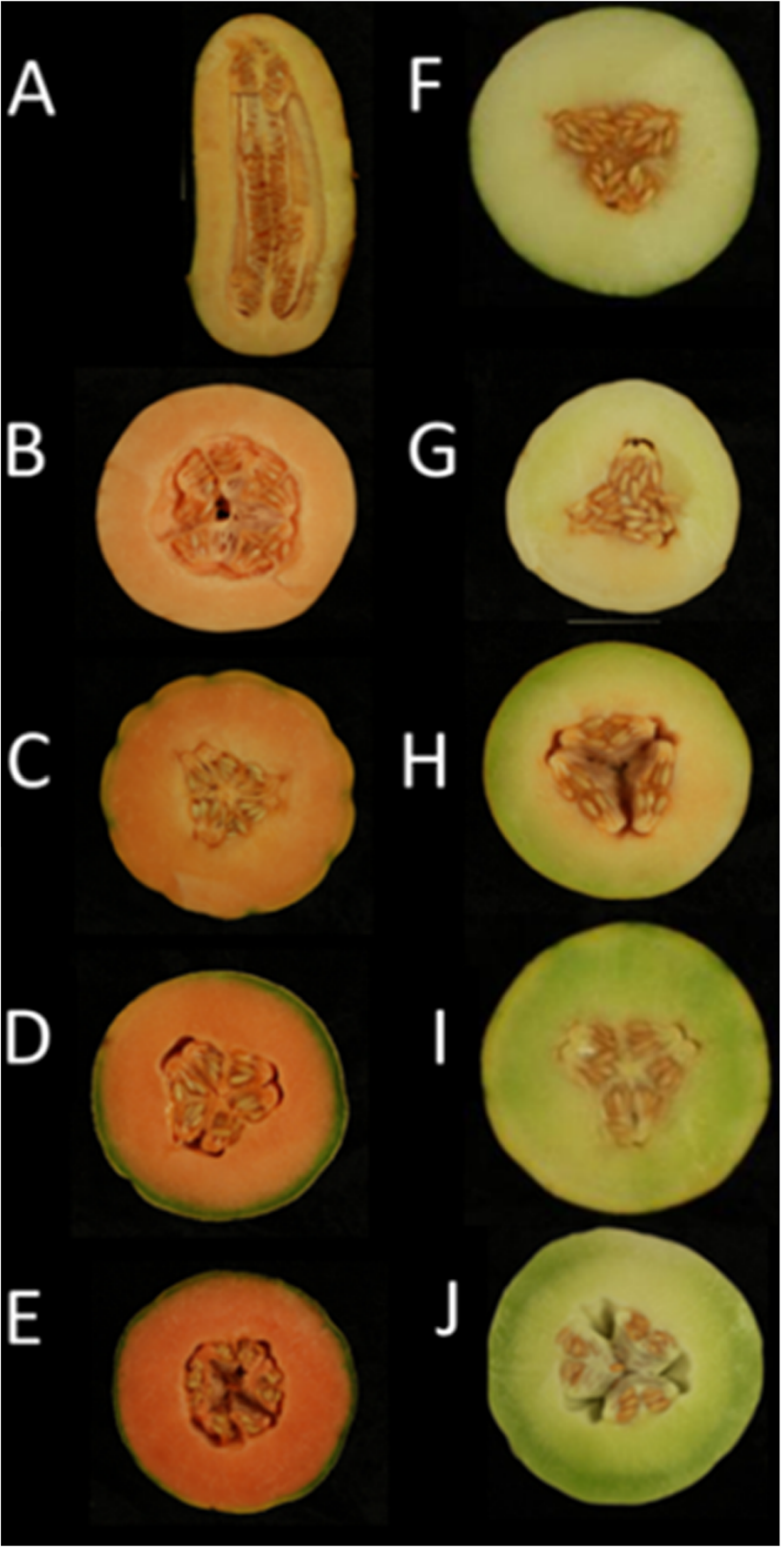
Representative fruit of 10 inbred lines cut open showing various flesh color phenotype. A-E: orange flesh phenotype, homozygous dominant *CmOr* encoding CmOR protein with a histidine at position 108. F-G: White and green flesh phenotypes, homozygous recessive *Cmor* encoding CmOR with an arginine at position 108. Accession names and taxonomic groups: (**a**) *PI 414723* (subspecies *agrestis*); (**b**) Indian Best, Chandalc; (**c**) *CEZ*, Cantalupensis (marketed as ‘Charentais’); (**d**) *Dulce*, Cantalupensis (marketed as Catalope); (**e**) *HP*, Cantalupensis, (marketed as ‘magenta-type’); (**f**) *Piel De Sapo*, Inodorus; (**g**). *NA*, Inodorus (marketed as ‘Canary Yellow’); (**h**). *Ein Dor,* Reticulatus; (**i**). *Noy Yizreel*, Cantalupensis; and (**j**): *Tam-Dew*, Inodorus (marketed as ‘Honey-Dew)’. All plants were field grown in the summer of 2012

Melon (*Cucumis melo*) is an economically important crop and has been subjected to intensive breeding programs for over a century [11]. Roughly 29.5 million tons of melon fruit were produced worldwide in 2013 [12]. Melon is a diploid (2n = 24) species with a relatively small genome size (estimated 450 Mb), which was recently sequenced and assembled [13]. Melon fruit flesh color is an important quality trait typically divided into three phenotypes: white, green, and orange. However, the color intensity may vary dramatically within these groups (Fig. 2). The orange fruit flesh phenotype is dominant over the non-orange phenotypes. The orange versus non-orange flesh color trait inheritance is controlled by a single gene termed *green-flesh,* which determines dominantly the accumulation of relatively high levels of β-carotene in orange flesh fruit [14]. Recently we reported that the melon’s *Or* gene (*CmOr*) governs the “*green-flesh”* trait [15]. OR, a plastid localized protein, increases carotenoids accumulation by inducing the biogenesis of chromoplasts with an enhanced sink strength [16, 17]. Several single nucleotide polymorphism (SNPs) distinguish between the *CmOr* alleles that dictate orange and non-orange fruit flesh colors, but only one of them alters an amino acid in the CmOR protein, an arginine at position 108 in white and green-flesh fruit is replaced by a histidine in orange flesh fruit. Functional proof for the role of this amino acid alteration in the phenotype determination was obtained by site directed mutagenesis followed by transgenic expression in *Arabidopsis* callus system [15]. A comparative transcriptome analysis of the two *CmOr* alleles in developing melon fruit could identify differentially expressed genes. Many of these genes are likely to be directly or indirectly associated with metabolic and cellular processes affected by *CmOr* allelic variation, or in other words, part of the gene network that is affected by *CmOr* allelic variation. This data will shed more light on *CmOr* function and mechanism of action.

Bulk segregant analysis (BSA) was established in 1991 as a method to detect markers in a specific genomic region by comparing two pooled DNA samples of individuals from a segregating population [18]. Within each bulk, the individuals are arbitrary for all traits except the trait or the gene of interest. The pooled individuals share the same genotype in the genomic area that surrounds the gene that distinguishes between the bulks. Coupling BSA with the high throughput RNA sequencing (RNA-Seq) has been shown to be an efficient tool for gene mapping and has been termed BSR-Seq [19, 20]. We hypothesized that comparing the transcriptomes of bulked melon F_3_ families, derived from a cross between orange and green fruited parental lines, with different flesh color, would identify differentially expressed genes (DEGs) that are associated with *CmOr* allelic variation.

In this study, we applied BSR-Seq to reveal metabolic and cellular processes associated with β-carotene accumulation under the control of *CmOr* allelic variation in orange and green flesh melon fruit. We show that BSR-Seq is an effective approach for gene discovery. Our results point to an association between the initiation of β-carotene accumulation and gene expression in the processes of photosynthesis, RNA and protein regulation, stress response, and interestingly sucrose metabolism that could be affected by *CmOr* allelic variation, or by variation in genes that are tightly linked to *CmOr*.

## Results

### The bulking process - phenotypes of the bulks and the parental lines

**Fig. 3:**
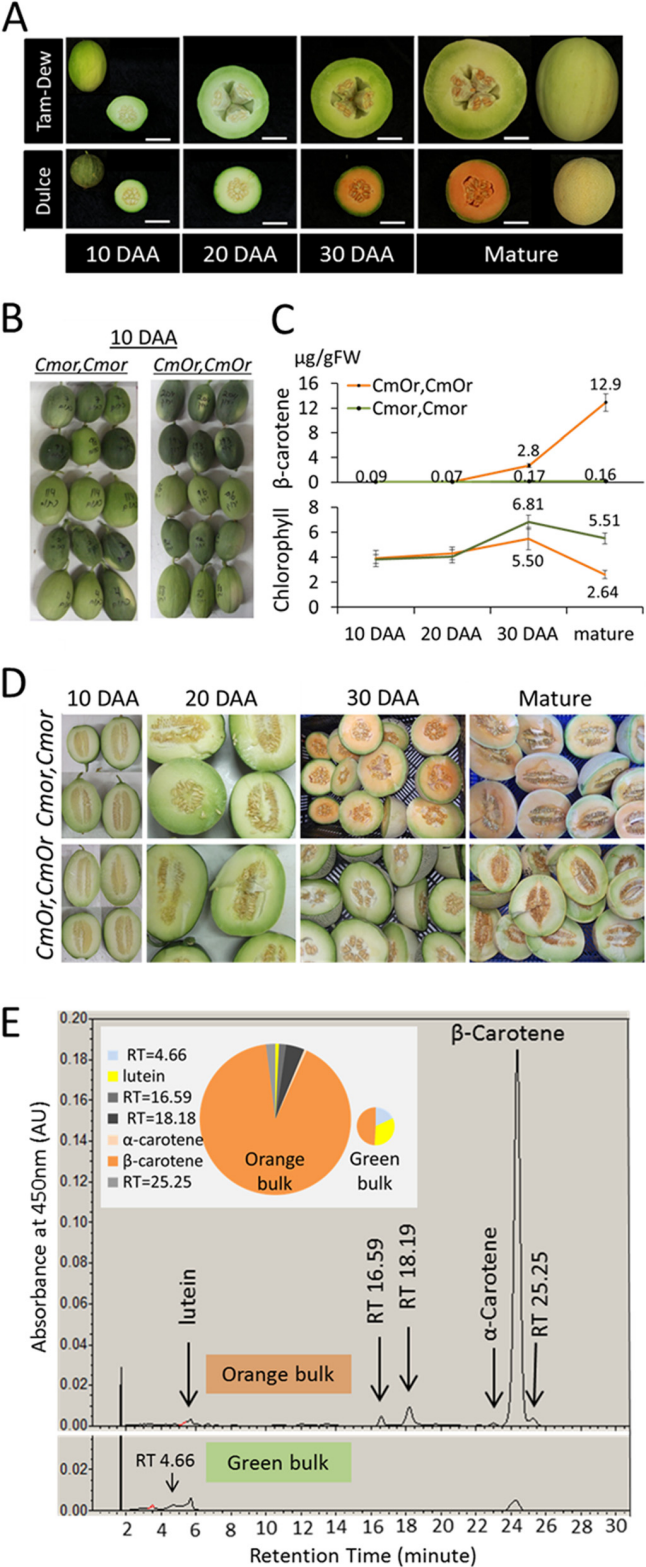
Phenotypic characterization of melon fruit bulks and parental lines. **a** Developing fruit of ‘Dulce*’* and ‘Tam-Dew*’*, the parental lines of the segregating population, at four developmental stages. Uncut fruits are shown at 10 DAA and at the mature stage. Bar = 5 cm; (**b**) 10 DAA fruitlets. Each three horizontal fruitlets belong to the same F_3_ family. Pictures of five out of the 25 families’ fruits comprising green (*Cmor,Cmor*) and orange (*CmOr,CmOr*) fruited bulks are depicted. Traits such as fruitlet rind color, fruitlet shape, striped or unstriped rind can be noticed in the pictures. Variation is evident within and between the F_3_ families and within the bulks but not between the compared ‘green’ and ‘orange’ bulks; (**c**) Accumulation of β-carotene and total chlorophylls (a + b) at four developmental stages. The values of β-carotene were obtained by HPLC analysis, the values of chlorophylls were obtained spectrophotometrically and they are all means ± SD of three biological repeats. Each repeat is constructed of 25 fruits, one from each of the F_3_families that comprise each of the bulks; (**d**) F_3_ fruit flesh color at four developmental stages. Color difference between the bulks became visually evident at 30 DAA; (**e**) Quantitative HPLC analysis of carotenoid content in the mature stage of each bulk: representative HPLC chromatograms of the elution profiles at 450 nm for each phenotype are presented. Lutein, α-carotene and β-carotene were identified according to their characteristic retention time (RT), distinctive spectra and comparison with authentic standards. Unidentified carotenoids are named by their characteristic RT. Pie-graphs sizes represent the relative total carotenoid content of the green and the orange mature fruit bulks measured at 450 nm. The inner partition represents the relative integrated peak area at 450 nm

We chose the segregating population originated from a cross between the orange flesh fruit ‘*Dulce*’ (‘*Dul*’) and the green flesh ‘*Tam-Dew*’ (‘*Tad*’) for constructing the bulks that were comparatively analyzed using BSR-Seq. In addition to fruit flesh color, ‘*Dul*’ and ‘*Tad*’ fruits differed in size, shape, rind darkness at 10 days after anthesis (DAA), rind color of the mature fruit, and netting on mature fruit peel (Fig. 3a). The parental lines also differed in the levels of total soluble solids (TSS), sucrose concentration, taste, aroma, rind width, rind hardness, and time to reach maturation, among other agronomical important traits. Selected bulked F_3_ families were phenotyped for these traits and except for the TSS levels and sucrose content of mature fruits no differences were found to distinguish between the mean values of the ‘green’ (*Cmor*/*Cmor*) and ‘orange’ (*CmOr*/*CmOr*) bulks. For example the average mature fruit weight of ‘*Dul’* was 938 g while ‘*Tad*’ fruit weighed 2218 gr (2.4 fold more). However, the average orange and green mature fruit weight of the bulked families (based on 75 fruits; 3 fruit of each of the 25 families in each bulk) weighed 1512 g and 1496 g respectively, showing insignificant differences in average fruit weight (Additional file 1: Figure S1 A and B). This demonstrated the effectiveness of the bulk approach to normalize differences between parental lines in traits that are unrelated to carotenoid accumulation, which is governed by *CmOr* allelic variation. As expected from such polygenic quantitative trait, the 25 families presented normal distribution around the mean (Additional file 1: Figure S1C).

Another example of the normalizing effect of the bulks on a trait that differs between parental lines was the number of days to flowering (as indicated by the first successful pollination). Like fruit weight, this trait is controlled by numerous genes since it is dependent on many factors such as plant growth rate, female flowering time, preferences of pollinators and stigma receptivity. While ‘*Dul*’ plant on average was successfully pollinated on May 15^th^ 2012, ‘*Tad*’ plant on average was successfully pollinated on May 23^rd^, exhibiting a substantial and significant (*P* < 0.01) eight days difference. However, the ‘successful pollination date’ of the ‘orange’ and the ‘green’ bulks were both averaged to May 19^th^ (18.95 and 19.24 on May, respectively), indicating again the trait normalizing attribute of the bulking approach.

The same bulking genetic effect was also evident for mono-genic traits, such as rind color of the young fruitlet, where young fruitlet dark green rind is dominant to light green [21]. Fruitlet rinds at 10 DAA were either dark green or light green (Fig. 3b). This trait segregated equally between bulks, independently of fruit flesh color and variation existed between and within the F_3_ families of both bulks. The dark green rind of the young fruitlet originated from ‘*Dul*’ (orange flesh parent) and is dominant over the light green rind that is derived from ‘*Tad*’ (green flesh parent). Out of the 25 families that were used to construct the orange-flesh bulk, 6 had light green rind, 7 had dark rind, and 12 segregated for this trait. Out of the 25 families that were used to construct the green-flesh bulk, 7 had light green rind, 5 had dark rind, and 13 segregated to this trait as expected for independent monogenic trait. Fruits in the segregating families were randomly chosen for each of the three replicates ensuring nearly similar representation of each phenotype within each bulk. Taken together, the bulk approach distinguished fruit flesh color and normalized differences in other unrelated traits between bulks. Thus, the transcriptome differences detected between the orange and green flesh fruit bulks were expected to be mainly associated with the effects of the *CmOr* gene.

Interestingly, we found a significant difference in mature fruit total solid soluble (TSS, Brix^0^) between the bulks. ‘*Tad*’, the green parent, had higher TSS levels (15.9 Brix^0^) than ‘*Dul*’ (13.9 Brix^0^), the orange parent (Additional file 1: Figure S1 H). These were not equalized by the bulking process and the ‘green’ bulk maintained significantly higher TSS (14.5 Brix^0^) than the ‘orange’ bulk (13.1 Brix^0^) (Additional file 1: Figure S1F-G).

### β-carotene and chlorophyll accumulation during fruit development

‘*Dul*’ fruit accumulates predominantly β-carotene in the mesocarp tissue [7]. Fruit flesh β-carotene levels of the ‘green’ and ‘orange’ bulks were measured by HPLC at four developmental stages: 10, 20, and 30 DAA and mature fruit (Fig. 3c). The fruit of the ‘green’ bulk contained only traces of β-carotene in all fruit developmental stages. The fruit of the ‘orange’ bulk started to accumulate β-carotene after 20 DAA, contained 2.8 μg per g of fresh weight (FW) at 30 DAA and reached the level of 12.9 μg g^−1^ FW upon maturation.

Chlorophylls levels during fruit ripening were also measured. The fruit of both the ‘orange’ and ‘green’ bulks contained 3.9 to 4.5 μg g^−1^ FW chlorophylls at 10 and 20 DAA, showing no significant differences (Fig. 3c). Furthermore, both bulks accumulated higher levels of chlorophylls at 30 DAA that declined toward maturation. However, the bulk of the green fruit contained higher levels of chlorophylls than the bulk of the orange fruit at 30 DAA (6.8 and 5.5 μg g^−1^ FW, respectively), and the difference became larger at the mature stage (5.5 and 2.6 μg g^−1^ FW, respectively).

Fruit flesh color within the bulks during the four developmental stages is shown in Fig. 3d and Additional file 1: Figure S1C. The color difference between the bulks was first visually noticed at 30 DAA (Fig. 3d), correlated with the accumulation of β-carotene as measured by HPLC. However, a slight difference in the fruit yellowness was clearly identified at 20 DAA by the Chroma-meter measurements, followed by a more dramatic difference in fruit redness, which was measured at 30 DAA and at the mature stage (Additional file 1: Figure S1D). Mature fruit color measurements of the parental lines, F_1_ hybrid and the bulks of segregants are shown in Additional file 1: Figure S1E.

The dramatic difference in β-carotene levels during fruit development was accompanied by different carotenoid composition in the bulks. The mature fruit of the ‘orange’ bulk contained mainly β-carotene (90.9 % of the total integrated peak area at 450 nm). Other detected carotenoids were lutein (0.75 % of the total integrated peak area at 450 nm), α-carotene (0.6 % of the total integrated peak area at 450 nm), and 3 other unidentified carotenoids with retention time (RT) 16.59, 18.19 and 25.25, comprising 1.5, 3.9 and 2 %, respectively, of the total integrated peak area at 450 nm (Fig. 3e). The mature fruit of the ‘green’ bulk had a 14.2 times lower total integrated peak area of detected carotenoids at 450 nm compared to the bulk of orange melon fruit. We were able to quantify the peak area of only three carotenoids of the bulk of green fruit: β-carotene (49.1 % of the total integrated peak area at 450 nm), lutein (32.8 %) and a third unidentified carotenoid (RT 4.66, 18.1 %) (Fig. 3e). We also identified traces of phytoene and ζ-carotene in the orange fruit bulk at their peak absorbance, 290 nm and 400 nm, respectively. These intermediate carotenoids were undetectable in the green fruit bulk (Additional file 1: Figure S2).

### SNPs analysis

**Fig. 4:**
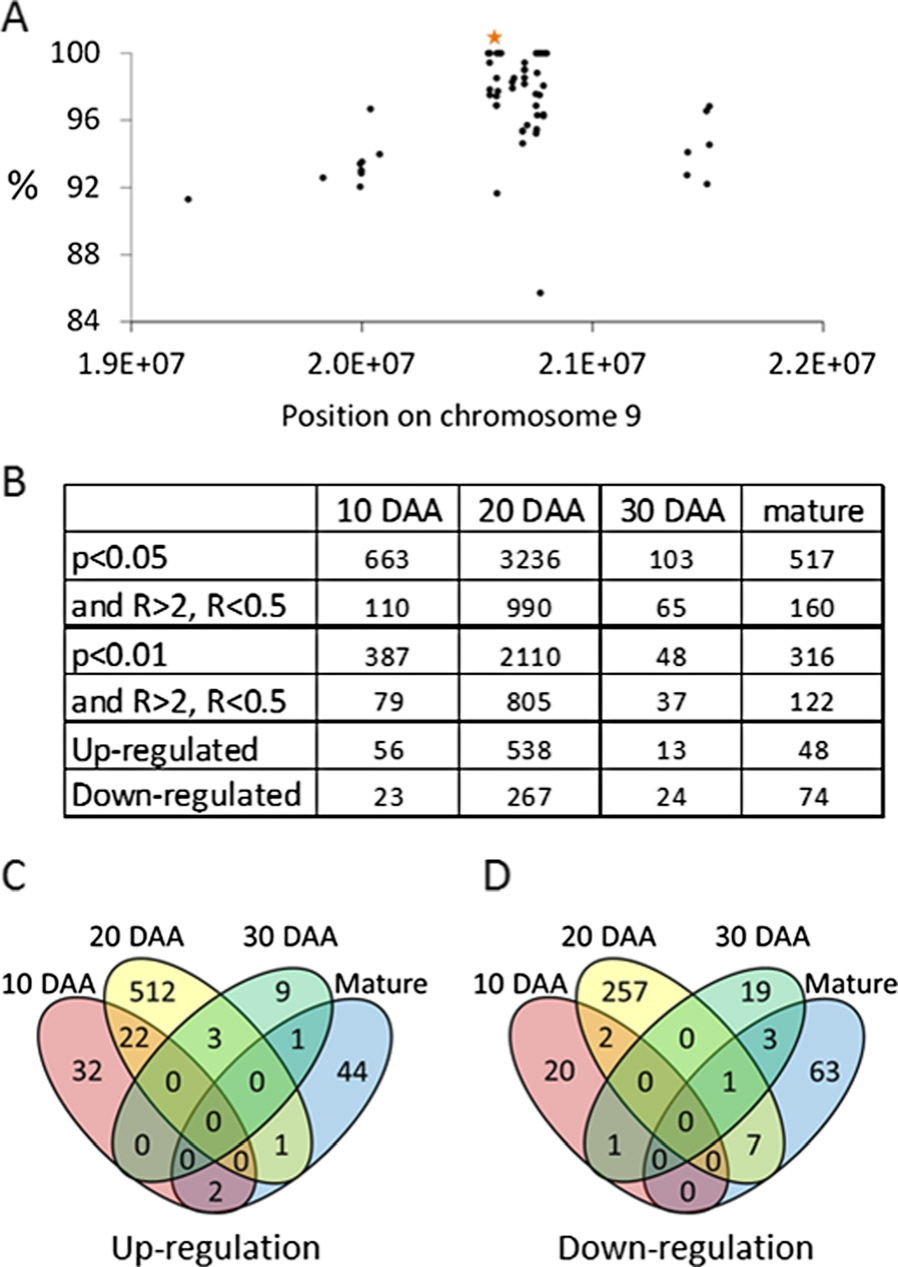
Bulk segregant transcriptome and SNPs analyses of melon fruit with different flesh color. **a** SNPs analysis of ‘green’ and ‘orange’ bulks of fruit identified only 64 SNPs (in genes with coverage higher than at least ten reads in each bulk and showing more than 90 % difference between bulks). All of identified SNPs surrounded *CmOr* gene in a range of 2,258,903 on chromosome 9. Each point in the figure represents one SNP. The X axis shows the location of each SNP on chromosome 9 and the Y axis represents the percentage of the polymorphic reads. Location of *CmOr* is marked with an orange asterisk; (**b**) The number of DEGs using two different adjusted *P* values (0.05 and 0.01) at the four analyzed fruit developmental stages. R is the ratio between mean RPKM (reads per kilobase, per million sequenced reads) of ‘orange’ bulk divided by mean RPKM of ‘green’ bulk; (**c**) and (**d**). Venn diagrams of up-regulated *P* < 0.01 and *R* > 2 (**c**) and down-regulated *P* < 0.01 and *R* < 0.5 (**d**) genes at the four fruit developmental stages

As described above, the bulks were constructed to minimize differences between parental lines that were not related to *CmOr* allelic variation. We were successful in doing so as revealed by the identification of only 64 SNPs between the ‘orange’ and the ‘green’ bulks. All SNPs were located in a physical proximity to *CmOr* in a region of 2,258,903 bp on chromosome 9 (Fig. 4a; Additional file 2: Table S2). These 2,258,903 bp, included 291 genes that were annotated and listed in Additional file 3: Table S3. Some of these genes may contribute to the differences between the bulks due to a genetic linkage with *CmOr* gene. The *CmOr* allelic variation of six SNPs that were recently reported [15], differentiated between the ‘green’ and the ‘orange’ bulks in 100 % of the reads (Fig. 4a, orange asterisk). Except *CmOr*, there was only one additional gene adjacent to *CmOr* that completely distinguished (100 %) between the bulks; MELO3C005486. This gene is homologous to a protein transporter that encodes for a pathogen-inducible nitrate/nitrite transporters in grapevine and in *Arabidopsis* [22] and is most probably not associated with carotenogenesis or chromoplasts biogenesis. Moreover, only 11 and 12 reads were recorded for the two SNPs identified within this gene (Additional file 2: Table S2).

### Comparative bulks transcriptome analysis

We used BSR-Seq of developing fruit mesocarp of the ‘green’ and the ‘orange’ bulks to identify cellular and metabolic processes affected by *CmOr* allelic variation. The 24 barcoded RNA-Seq libraries were sequenced on a single lane of an Illumina HiSeq 2000 run. A total of between 3.5 and 8.5 million reads from each library were produced with an average of 78.4 % of them that were mapped to the melon genome. We preformed statistical analysis to identify genes that were differentially expressed between the ‘green’ and the ‘orange’ bulks at the different fruit developmental stages (Fig. 4b). A total of 79, 805, 37 and 122 genes were differentially expressed at 10, 20, 30 DAA, and the mature stage, respectively (Additional file 4: Table S4). Noticeably, the largest number of DEGs were observed at 20 DAA, the stage when slight flesh color change could first be measured by the Chroma-meter (Additional file 1: Figure S2C).

At the two earlier developmental stages (10 and 20 DAA), before significant amounts of carotenoids start to accumulate, most of the up-regulated genes were in the ‘orange’ bulk (67 %: 594 out of 884), while during the two later stages (30 DAA and mature fruit) most of the down-regulated genes were in the ‘orange’ bulk (62 %: 98 out of 159). The vast majority of the DEGs were uniquely altered at a particular fruit developmental stage (Additional file 4: Table S4). However, 26 genes were up-regulated in the ‘orange’ bulk in two consecutive stages and 6 were down regulated (Fig. 4c–d) (Additional file 5: Table S5). Only one gene (MELO3C005241, a microtubule binding protein) was differentially down-regulated in three consecutive stages (Fig. 4c). Although this gene is co-gentically linked to *CmOr* on chromosome 9 its effect on carotenoids accumulation needs further studies.

### qRT-PCR verification of BSR-Seq differentially expressed genes

*CmOr* allelic variation caused transcriptomic changes in fruit during maturation. In order to validate the accuracy of the RNA-Seq data, we performed qRT-PCR study of 30 selected DEGs along with *CmOr*. These genes were selected according to their expression patterns and expression ratio between the bulks of developing fruit. For each fruit developmental stage, we chose DEGs displaying the highest ratio between ‘orange’ and ‘green’ bulks. The chosen DEGs expression at the relevant stage of fruit development was substantially higher than in the other stages. We measured by qRT-PCR the relative expression of these selected DEGs in the fruit flesh of the bulks and of the population parental lines, ‘*Dul*’ (orange) and ‘*Tad*’ (green). The relative expression of each DEG was measured by qRT-PCR analysis at the developmental stage in which the differential expression was first observed. In all the examined DEGs, the qRT-PCR results were in accordance with the RNA-Seq of the bulks (Additional file 1: Figure S3A). The correlation coefficient (r) of the examined DEGs was 0.94 showing highly significant correlation between relative and digital expression (Additional file 1: Figure S3B). When parental lines were included in the analysis, there was one gene (MELO3C005487) that showed a complete opposite relative expression pattern and additional four genes (MELO3C008862, MELO3C005502, MELO3C001914 and MELO3C008287) that did not show different relative expression between parental lines (Additional file 1: Figure S3A). This can be best explained by the parental lines different maturation paces, which were normalized in the bulks.

### Cellular processes affected by *CmOr*

**Fig. 5:**
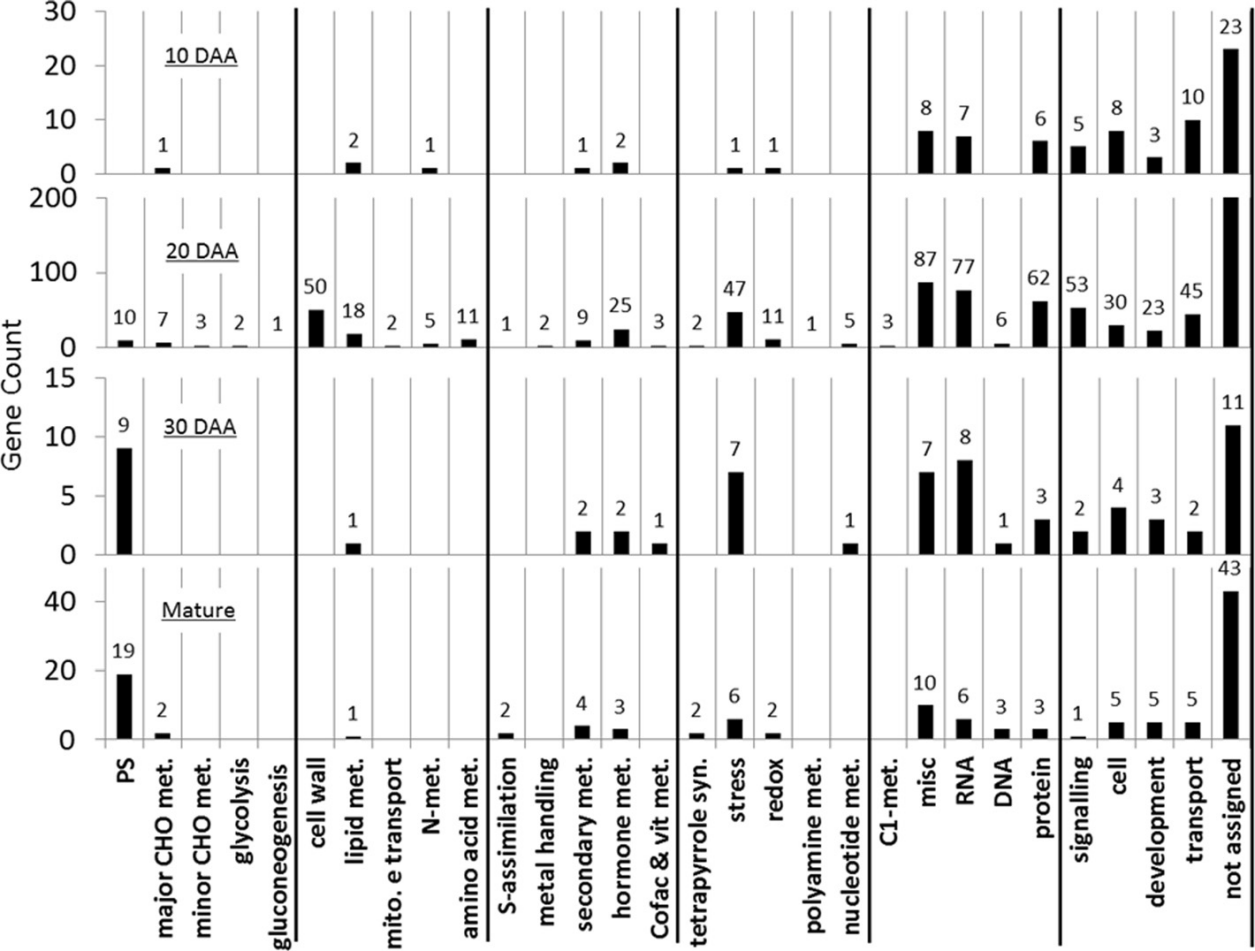
Cellular processes affected by *CmOr*. Gene counts according to their MapMan bin-code name of cellular processes. Each bar represents the number of DEGs between the ‘green’ and the ‘orange’ bulks at (from top to bottom) 10, 20 and 30 DAA and at mature fruit stages. An adjusted *P* value of 0.01 was used to detect DEGs at 10, 20 DAA and the mature fruit stages, while for 30 DAA we used an adjusted *P* value of 0.05. PS = Photosynthesis; CHO = carbohydrates; met = metabolism; syn = Synthesis; mito. E transport = mitochondrial electron transport; cofac & vit = cofactors and vitamins; C1 = one carbon

The DEGs at the different fruit developmental stages were categorized into functional groups using MapMan [23]. This analysis revealed that the distribution of DEGs in most functional classes varied depending on the fruit developmental stage (Fig. 5). The relatively enriched functional groups were those involved in transport, cell, RNA and protein processes at 10 DAA, RNA, protein, and signaling at 20 DAA, photosynthesis, RNA, and stress at 30 DAA and photosynthesis at the mature fruit stage (Fig. 5). The unclassified group of DEGs, distributed equally between melon fruit developmental stages.

*Photosynthesis related genes:* A total of 10, 9 and 19 of the DEGs were clustered by MapMan analysis as photosynthesis related at 20 DAA, 30 DAA, and the mature fruit stages, respectively. The photosynthesis related cluster is the most abundant one in the two later developmental stages (Fig. 5). As shown in Fig. 3c chlorophyll levels were similar in the ‘orange’ and the ‘green’ bulks during the two earlier stages and differed at the two later stages. Furthermore, the most notable shift in chlorophyll levels during fruit development was observed in the ‘orange’ bulk between 30 DAA and the mature stage (more than 2-fold reduction from 5.5 to 2.64 μg/gFW tissue). In accordance, all the photosynthetic DEGs were down regulated in the ‘orange’ bulk during these later fruit ripening stages. These genes include structural genes of photosystem I and II, as well as other electron carrier and genes encoding for Calvin cycle enzymes (Additional file 6: Table S6).

For example, the expression level of MELO3C000130, an ortholog of the Arabidopsis large subunit of RUBISCO (ATCG00490), was 4.3 times higher in the ‘green’ bulk than in the ‘orange’ bulk at the mature stage. Another gene was MELO3C01967, an ortholog of a light-harvesting complex II subunit (AT1G29930), which transfers absorbed light energy to the reaction center of photosynthesis. Its expression level was doubled in the ‘green’ bulk at the mature stage. A third example was MELO3C008731, an ortholog of AT4G12800 that encodes for subunit L of photosystem I reaction center in Arabidopsis. Its expression was 2.6 higher in the ‘green’ bulk than in the 'orange’ bulk at the mature stage. The down-regulation of these genes in the orange fruits was concomitant with the difference in chlorophyll contents between the orange- and green-flesh fruits (Additional file 6: Table S6).

*RNA related genes*: A total of 7, 77, 8 and 6 DEGs were clustered as RNA related at 10, 20, 30 DAA and in mature fruit, respectively. At 20 DAA, RNA was the most abundant functional group. Noticeably, among the 77 RNA-related DEGs, 75 were transcription regulators. These differentially expressed regulators probably played a role in the transcriptional differences of a large number of genes between the ‘orange’ and ‘green’ bulks at 20 DAA, the time point of the initiation of fruit flesh color transition. It is likely that a regulatory network of transcription was activated in the *CmOr* orange bulk fruit. Interestingly 11 differentially expressed transcription factors at 20 DAA belonged to the APETALA2/ethylene-responsive element binding protein family (AP2/EREBP). The AP2/EREBP supergene family is known to be involved in the regulation of stress related genes [24] (Additional file 6: Table S6).

*Stress related genes:* The *Or* gene has been previously associated with photo-oxidative stress responses in cauliflower (*Brassica oleracea*) and the *Or* mutant seedlings during de-etiolation showed higher expression levels of ROS-responsive genes [25]. Moreover, in sweet potato (*Ipomoea batatas)* callus system, overexpression of *IbOr* was associated with increasing of salt stress tolerance [26]. A total of 1, 47, 7, 6 DEG were clustered as stress related at 10, 20, 30 DAA and in mature fruit, respectively. At 20 DAA, 19 of these DEGs were related to heat stress, 13 to biotic stress, and 9 to drought/salt stress response, 2 to wounding/touch stress, and 4 genes to unassigned stress response (Additional file 6: Table S6).

Abscisic acid (ABA) is a product of the carotenoid metabolic pathway (Fig. 1) and its production is regulated by environmental cues [27]. ABA is known to regulate genes in response to environmental changes, in particular osmotic stress as reviewed in [28]. MELO3C005129, an ortholog of xanthoxin dehydrogenase (AT1G52340; *ABA-2*), encodes a cytosolic short-chain dehydrogenase converting xanthoxin to ABA-aldehyde during ABA biosynthesis. Its expression was higher at all the developmental stages in the ‘orange’ bulk, statistically significant and above the 2 fold cutoff at 30 DAA and in mature fruit (2.8 fold and 4 fold higher, respectively) (Additional file 1: Figure S4).

*Protein metabolism and processing related genes*: A substantial number of DEGs were assigned as genes associated with protein-related processes. A total of 6, 62, 3, and 3 DEGs were clustered as protein related at 10, 20, 30 DAA and in mature fruit, respectively (Fig. 5). At 20 DAA, the stage when major transcript differences between the ‘orange’ and the ‘green’ bulks were noted, 25 genes were assigned to protein degradation, 27 were assigned to posttranslational protein modification (out of which 15 were kinases), 3 to protein targeting and 3 to protein synthesis (Additional file 6: Table S6).

### Changes in genes involved in carotenoid metabolism

**Fig. 6:**
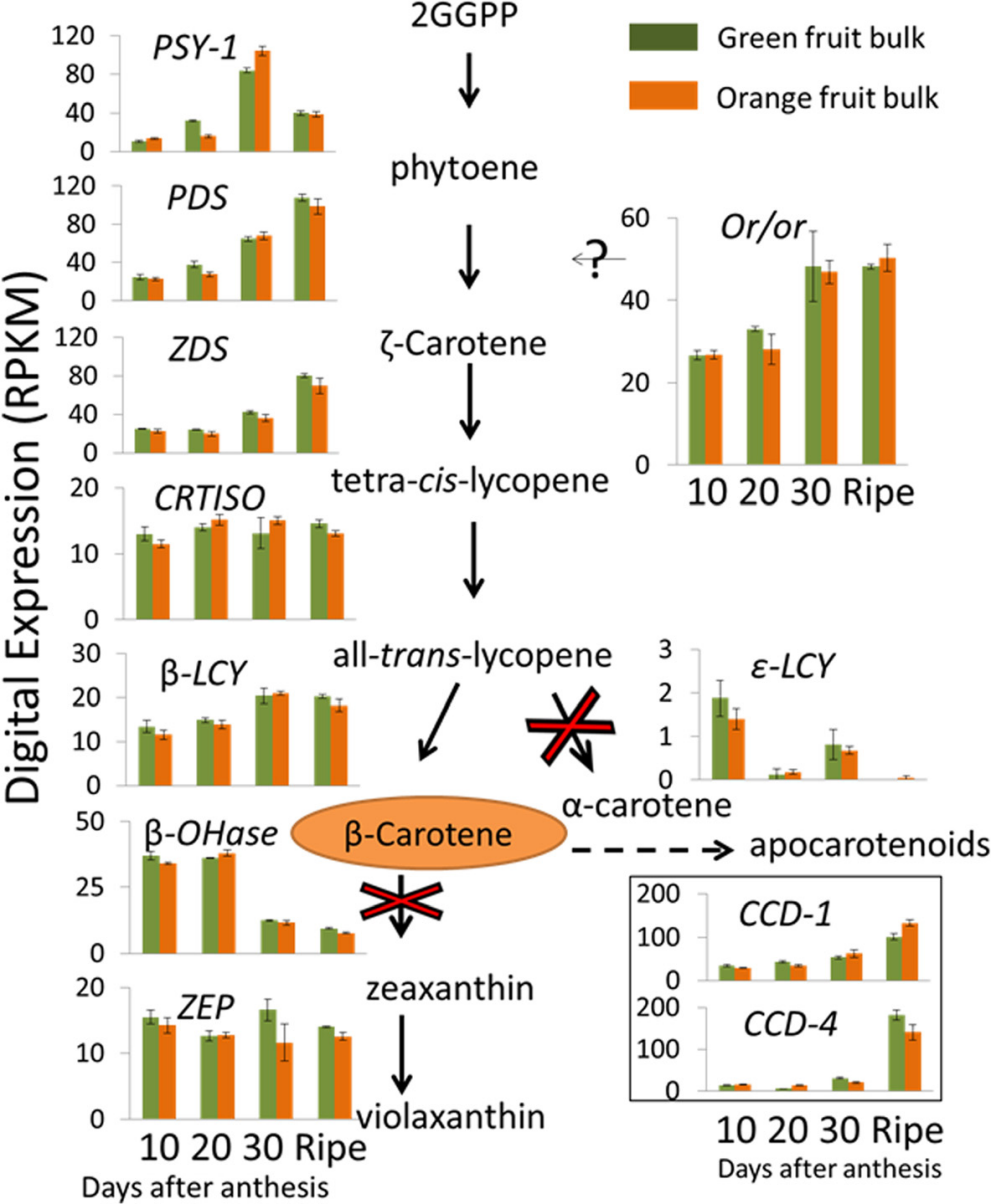
Expression of carotenogenesis genes. Each bar is the average RPKM of three biological repeats at each fruit developmental stage. Error bars represent standard error of the mean. When there was more than one melon gene annotated, we chose to present the gene with the highest expression. The gene IDs are (https://melonomics.net) *PSY-1*, MELO3C025102; *PDS,* MELO3C017772; *ZDS*, MELO3C024674; CRTISO, MELO3C016495; β-LCY, MELO3C020744; ε-LCY, MELO3C004633; β-OHase, MELO3C014945; *ZEP*, MELO3C020872; CCD4, MELO3C016224; CCD1, MELO3C023555*;* and *CmOr*, MELO3C005449

Interestingly, the genes annotated to encode for enzymes in the carotenoid biosynthesis pathway were expressed similarly in both bulks (Fig. 6). Clearly, the transcript levels of carotenogenesis genes alone could not explain the higher carotenoid accumulation in the fruit flesh of the ‘orange’ bulk. However in the ‘orange’ bulk, where carotenoids are accumulated in the fruit, carotenogenesis genes expressions seem to schedule the accumulation and to regulate the carotenoid composition. PSY-1 and PDS activities are responsible for carotenoid levels [1, 29]. The increased expression of *PSY-1* and *PDS* was associated with the enhanced carotenoids levels at 30 DAA and mature stage of orange fruits. The very low transcript level of *ε-LCY* together with higher transcript level of *β-LCY* might direct the metabolic flux toward the production of β-carotene rather than α-carotene. *β-OHase* was down-regulated between 20 and 30 DAA and its low expression continued until the mature stage. The low transcript levels might reduce further modification of β-carotene into zeaxanthin and explain the dominance of β-carotene in the melon fruit flesh carotenoid composition.

Similarly, genes upstream of the carotenogenesis metabolic pathway in the MEP pathway were not differently expressed between the bulks (Additional file 1: Figure S5). Thus, MEP gene expression is not affected by *CmOr* allelic variation and could not explain the ‘orange’ bulk phenotype. Similarly to the structural carotenogenesis genes, MEP genes were up-regulated at 30 DAA and at the mature stage of both bulks in correlation with the time of carotenoid accumulation in the ‘orange' bulk. *DXS* (MELO3C014965), which encodes the enzyme synthesizing 1-deoxy-D-xylulose-5-phosphate (DXP), was 5.9 fold higher in the mature fruit comparing to fruits at the earlier stages (Additional file 1: Figure S5). *DXR* (MELO3C026292), the next gene of the pathway, was also up-regulated during the later fruit developmental stages (Additional file 1: Figure S5). The next enzymatic step is the synthesis of GGPP, the building blocks of phytoene by geranylgeranyl reductase (GGR; MELO3C013320). *GGR* was also up-regulated in the later fruit maturation stages (Additional file 1: Figure S5). We did not find significant changes of the downstream genes of the metabolic pathway other than up-regulation of *ABA-2* in the ‘orange’ bulk as described above (Additional file 1: Figure S4).

### Sugar metabolic pathway analysis

**Fig. 7:**
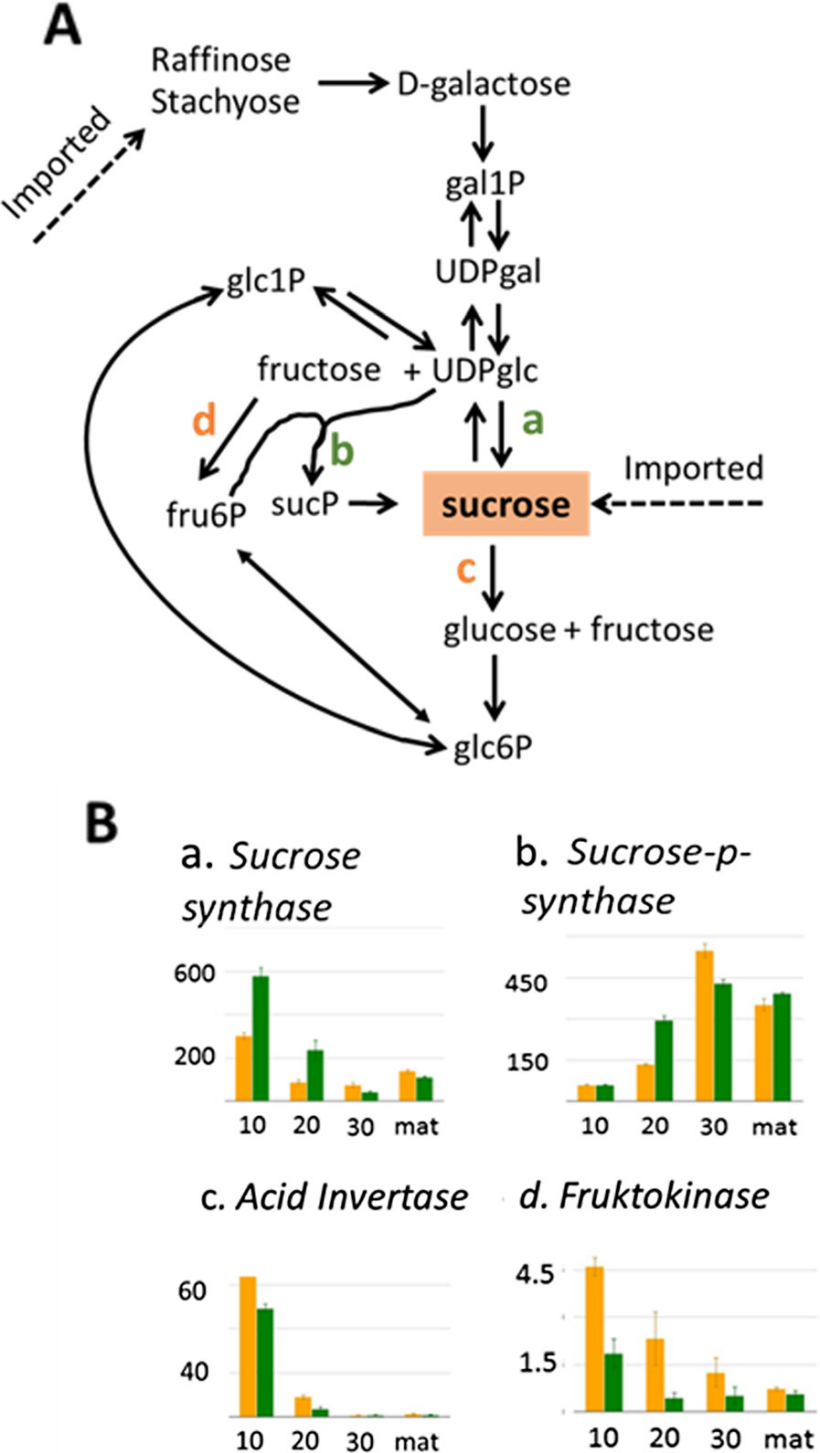
DEGs related to sucrose metabolism. **a** 20 DAA DEGs placed on metabolic pathways leading to sucrose accumulation in melon fruit; sucrose synthase (*a*) and sucrose-p-synthase (*b*) leading to sucrose accumulation are up-regulated in the ‘green’ bulk (*green letters*) while acid invertase (*c*) and fructokinase (*d*), leading to turnover of sucrose and sucrose precursors are up-regulated in the ‘orange’ bulk (*orange letters*). Unmarked arrows indicate genes with similar expression levels in the ‘orange’ and the ‘green’ bulks. This schematic pathway was modified after [33]. Glc-glucose, gal- galactose, fru-fructose, suc-sucrose, P-phosphate; (**b**) Expression pattern of the four DEGs marked in A during fruit development (10, 20, 30 DAA and mature fruit). The genes IDs are (https://melonomics.net): *a* MELO3C015552, *b* MELO3C010300, *c* MELO3C005363, and *d* MELO3C014574

We used plant MetGenMAP [30], a web-based bioinformatics tool, to search for significantly altered metabolic pathways. The significantly changed pathways included galactose and sucrose degradation (*P* value = 0.017, 0.028 respectively) at 20 DAA. In sweet melon, stachyose and raffinose are translocated to the fruit and sucrose accumulation is associated with developmentally regulated transcriptional changes of sugar metabolism genes in the fruit sink itself [31]. Our results indicated significant changes in expression of genes related to sugar metabolism at 20 DAA. We marked the DEGs that were pointed out by MetGenMAP on the previously elucidated metabolic pathway leading to melon fruit sucrose accumulation (Fig. 7). The two genes leading directly to sucrose synthesis, *SUSY* (which acts in both directions) and *SPS* were up-regulated in the ‘green’ bulk at 20 DAA, while genes degrading sucrose (*invertase*) and shifting the metabolic flux away from sucrose (*fruktokinase*) were up-regulated in the ‘orange’ bulk at 20 DAA (Fig. 7, Additional file 4: Table S4). The causal gene for these significant changes could be either *CmOr* or another gene genetically linked to *CmOr*.

### Sugars levels and composition at 30 DAA and at the mature fruit stage

Sucrose accumulation in melon fruits is a developmentally regulated process. Previous studies showed that young developing melon fruits do not accumulate sucrose [32, 33] and that sucrose is accumulated following transcriptional changes in fruit sugar metabolism genes [31]. The transcriptional changes in sugar metabolism found here are consistent with the observation that mature fruit TSS was higher in the ‘green’ bulk. Since numerous previous studies showed strong correlation between mature melon fruit TSS and sucrose levels [34–36], we analyzed sugar content and composition at the late fruit developmental stages, when melon fruit accumulate sucrose [31, 32].

**Fig. 8:**
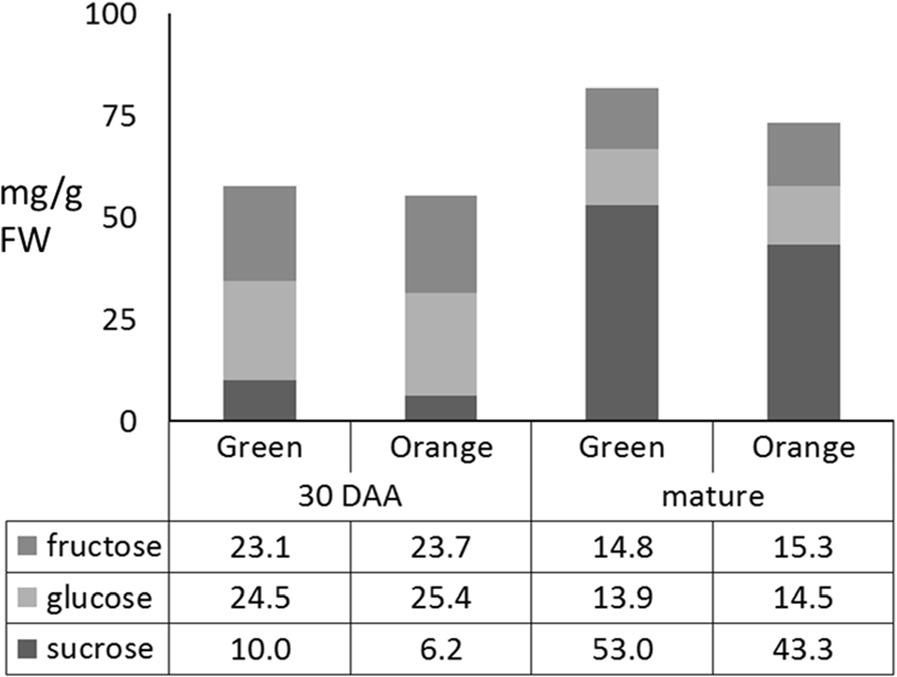
Melon’s sugars content. Sucrose, glucose and fructose content in fruit flesh were measured by HPLC at 30 DAA and in mature bulks of orange and green fruits. Each bar represents the mean of 75 fruits; three fruits from each of the 25 families comprising each bulk. Differences in sucrose levels are significant in both developmental stages (*P* < 0.05)

Expectedly, quantification of soluble sugars by HPLC at 30 DAA and at the mature stage indicated that sucrose levels significantly increased from 30 DAA to the mature stage (*P* < 0.05), partially at the expense of glucose and fructose levels (Fig. 8). Comparison of sugars levels between the bulks at each analyzed developmental stage, indicated that the differences in TSS were indeed due to the sucrose levels that were significantly higher in green fruit than in orange fruit at both stages (10 vs. 6.25 and 52.98 vs. 43.32 mg/g FW at 30 DAA and at the mature fruits, respectively), while glucose and fructose differences were insignificant (Fig. 8).

## Discussion

### BSR-Seq approach for the identification of genes and cellular processes that are associated with traits of interest

BSR-Seq is a straightforward method for mapping monogenic traits. Using this approach, an early study mapped a characterized maize mutant *glossy3* to the previously known locus at an interval of ~2 Mb. The only down regulated gene located in this interval in the mutant bulk was shown to be the *glossy3* [19, 20].

Using BSR-Seq of ‘green’ and ‘orange’ bulks, derived from a cross between ‘*Dul*’ (orange fruit flesh) and ‘*Tad*’ (green fruit flesh), we demonstrated the competence of this method to investigate the specific transcriptomic effects of a single mutation, similarly to the accepted use of near-isogenic lines. BSR-Seq approach was effective in normalizing phenotypes (Fig. 3 and Additional file 1: Figure S1) and genotypes (Fig. 4a) that differentiate between the parental lines but are unrelated to fruit flesh color, our trait of interest. Thus, the BSR-Seq approach led us to identify metabolic and cellular processes that are associated with the *CmOr* allelic variation. The transcriptome analysis suggested an activation of transcription regulation and of protein metabolism at the initiation of β-carotene accumulation. Furthermore, BSR-Seq analysis of mature fruits suggested a loss of the photosynthetic apparatus in orange but not in the green fruits. BSR-Seq analysis of fruitlets at 20 DAA indicated a later initiation of the sucrose accumulation stage in the orange fruit compared to the green fruit. We were able to link the two latter transcriptional differences to the physiological differences in mature fruit: the orange high β-carotene accumulating fruit had lower chlorophyll and lower sucrose levels compared to the green low β-carotene accumulating fruit.

Our results also demonstrate that BSR-Seq experimental design is indeed an advantageous tool for gene discovery. The identity and the role of *CmOr* in determining fruit flesh color in melon would be discovered using the experimental design as demonstrated with the SNPs analysis between bulks. Using BSR-Seq of orange and green fruited F_3_ families, we identified only two tightly linked genes that completely differentiated between these bulked phenotypes (Fig. 4a). It is most likely that by adding more F_3_ families within each bulk one would raise the probability to identify *CmOr* alone due to recombination events. However, for breeding purposes, a marker in close proximity to the genes of interests can probably be found by using even less F_3_ families in a smaller and cheaper experimental design.

### A global transcriptional view of cellular processes associated with CmOr allelic variation

The phenotype of *BoOr* mutant was first described in 1975 [37]. The *BoOr* mutated gene triggers the biogenesis of chromoplasts, which serve as metabolic sinks for carotenoid accumulation [16, 17]. Recently, Zhou et al. [38] showed that the wild-type Arabidopsis OR protein (AtOR) directly interacts with PSY and post-transcriptionally regulates PSY enzymatic activity as a mechanism by which AtOR boosts carotenogenesis in plastids. However, the mechanisms underlying the OR-regulated chromoplast biogenesis and its associated cellular processes are still not fully understood.

We performed comparative transcriptome analysis in hypothesis driven as well as in hypothesis driving means. The hypothesis driven approach included searching for changes in the expression of expected candidate genes (e.g., carotenogenesis genes). This approach revealed that *CmOr* did not regulate carotenogenesis genes expression, similarly to what had been previously found in cauliflower [39]. However, by clustering DEGs and studying the cluster annotations in a hypothesis driving manner, we found that *CmOr* allelic variation affected the expression of photosynthetic genes expression; was associated with protein metabolic processes; RNA regulation; and was correlated with cellular stress responses.

### *CmOr* association with photosynthetic genes

The most notable phenotype governed by *CmOr* allelic variation was a drastic increased level of β-carotene in the orange ripe fruit mesocarp (Fig. 3c and Additional file 1: Figure S2). The increasing carotenoid accumulation in the ‘orange’ bulk was associated with a decrease in chlorophyll levels (Fig. 3c), Lower chlorophyll levels together with down-regulation of photosynthetic and chloroplast associated genes in the ‘orange' bulk (Additional file 6: Table S6), may indicate chloroplast degradation or chloroplast to chromoplast transition. Transition from chloroplast to chromoplasts has been well described during tomato fruit development [40], yet this process had never been associated with melon fruit development, where biogenesis of chromoplasts directly from non-colored plastids has been hypothesized [15].

### *CmOr* association with protein post-translational regulation

While direct interaction of AtOR and AtPSY was recently described [38], our results suggest that additional post-transcriptional protein regulation was associated with *CmOr*. At 20 DAA, 62 DEG were related to protein modifications, including genes whose products are involved in protein degradation, protein folding and post-translational modifications, such as glycosylases, phosphorylases and carboxylases (Additional file 6: Table S6). The different expression of these genes was measured at the stage when color phenotypic difference was first seen (Additional file 1: Figure S1D). Further investigation of the associations of these genes with carotenoid accumulation in melon broad germplasm or in other fruit species will add new information towards our understanding of how *CmOr* regulates massive carotenoid accumulation.

### *CmOr* association with RNA regulation

Phytoene biosynthesis and desaturation, the first two steps of carotenogenesis are catalyzed by PSY and PDS (Fig. 1). Arabidopsis *PSY* and *PDS* were shown to include ATCTA *cis* acting element which are bound by the AtRAP2.2 transcription factor, a member of the APETALA2/Ethylene-responsive element binding factors (AP2/ERF) gene family [41–43]. Here we show that *CmOr* allelic variation is associated with differentially expressed RNA regulation genes, 11 of them belong to the AP2 gene family. However, we did not record differential *PSY* or *PDS* expression between the bulks. Arabidopsis *PSY* was also shown to be directly regulated by transcription factors of the phytochrome interacting factors (PIFs) which belong to the Basic Helix-Loop-Helix protein family (bHLH) [44]. Our data shows 11 bHLH transcription factors differentially expressed in association with *CmOr* allelic variation. *PSY1* promoter in tomato fruit was shown to directly interact with the MADS-box transcription factor RIPENING INHIBITOR [45].

The above examples suggest a complex role of transcription factors in regulating carotenogenesis. Still, very little is known about specific transcription factors which putatively play a role in carotenoid accumulation in melon and in other fleshy fruits. The data we describe here, of 75 deferentially expressed transcription factors at 20 DAA (Additional file 6: Table S6), may be used in the future to search for such factors. It is reasonable to assume that some of these transcription factors are mediators of chromoplast biogenesis, which is the major known role of the OR protein [16, 17].

### *CmOr* association with cellular stress responses

At 20 DAA, 47 DEGs were clustered as stress related (Fig. 5, Additional file 6: Table S6). The stress related and ABA synthesis DEGs (Additional file 1: Figure S4) probably did not act to promote a stress response since they were measured in green and orange melon fruits of vivid and well irrigated plants. This difference could result simply from the higher availability of ABA precursors (Fig. 1) or from yet unknown roles of these genes in fruit carotenoid accumulation. Alternatively, these DEGs might suggest a regulatory role of *CmOr* in controlling stress related genes.

In an evolutionary perspective, carotenoids were probably first evolved for their fundamental roles in photosynthesis. Later, they gained new adaptive roles as precursors of land plant hormones ABA and strigolactones (SL) [1]. In the later evolution of angiosperms, carotenoids were recruited to serve as pigments of flowers and fruits and their apocarotenoids derivatives were evolved to act as visual and volatile signals to attract pollinating and seed dispersal agents [46]. The *Or* gene, which functions as a regulator of carotenoid accumulation, is conserved throughout the plant kingdom, including primitive vascular plants i.e., the lycophyte *Selaginella moellendorffii,* the bryophyte *Physcomitrella patens* [15] and even in the more primitive unicellular green alga *Chlamidomonas* [17]. While the *Or* gene could had gained a totally new function in recent evolutionary times, a parsimonious possibility might suggest that *Or* serves as a regulatory gene of the carotenoid metabolic pathway leading to ABA and SL production. ABA and SLs are related to plant environmental stress responses such as drought and salinity [28, 47].

In fruits of well irrigated flourishing plants the putative role of *Or* in mediating stress response through carotenoid derived hormones seems to be phenotypically meaningless. However, this role can be clearly observed at the transcriptional level (Fig. 5 and Additional file 6: Table S6). We assume that the transcriptional stress response documented in our results may hint not only the ancestral role of *Or* but also to its role in other plant tissues.

### *CmOr* association with protein metabolism and processing

Sixty two of the 74 DEGs that were assigned as genes associated with protein-related processes were discovered at 20 DAA (Fig. 5). Forty seven, out of these 62 DEGs, were up-regulated in the ‘orange’ bulk, suggesting active protein metabolism in the orange melon fruit in comparison to green fruit. MELO3C004635, which was clustered as posttranslational modification kinase and annotated as mitogen-activated protein kinase (MAPK), was expressed 3.3 times higher in the ‘orange’ bulk. MAPK in leaves of maize plants was shown to be involved in ABA induced antioxidant defense reaction [48]. Two other kinases, which were up-regulated in the ‘orange’ bulk at 20 DAA, were MELO3C019919 and MELO3C026658 (3.09 and 2.37 fold higher than in the 20 DAA ‘green’ bulk, respectively) that possess a leucine-rich repeat motif. This motif, which is present in proteins of diverse functions, provides proteins with a flexible structure to facilitate protein–protein interactions [49].

#### CmOr allelic variation did not alter carotenogenesis gene expression

Transcriptional regulation of carotenoid metabolic pathway genes has been shown to be an important mechanism in controlling carotenoid levels in various plant species and tissues. In white flesh loquat mesocarp, low carotenoid content is associated with lower expression levels of *PSY1*, *β-LCY,* and *β-OHase* in comparison with their levels in orange flesh cultivar [50]. In marigold petals, the variation of carotenoid levels is attributed to MEP and carotenogenesis gene expression [51]. In tomato fruit, *PSY1* gene expression is closely associated with carotenoid levels during development [52]. Degradation processes has also been associated with fruit carotenoid accumulation, for example in peach fruits, where lower carotenoid levels in the white cultivars are associated with higher transcript levels of *CCD4,* which cleaves carotenoids [53].

Here we show the association between *PSY1* expression and β-carotene accumulation in developing orange colored fruits (Fig. 6). Surprisingly, the bulk of green fruit that do not accumulate carotenoids during fruit development exhibited the same fruit development associated expression pattern of *PSY1*. Similar to *PSY1*, almost all the genes leading to the formation or degradation of β-carotene, including *DXS, DXR, GGR, CmOr, ZDS* and *PDS* and *CCD*, were up-regulated during green fruit maturation. Their expression patterns were in good association with carotenoid accumulation in the orange bulk fruits, however, the *CmOr* recessive allele of the green fruit was not capable to induce massive carotenoid accumulation and ‘green’ bulk fruits did not accumulate carotenoids (Fig. 6 and Additional file 1: Figure S4). This could happen due to a rapid carotenoid turnover, due to an inability to form proper sink structure for stable storage [54], due to an inability to interact with PSY-1 [40] or due to another yet unknown mechanism.

The transcriptional changes of carotenogenic genes during orange melon fruit development also explain the predominant β-carotene accumulation in melon fruit; *β-LCY* was up-regulated and *ε-LCY* was down regulated, channeling the metabolic flux away from the α-carotene/lutein branch and towards the β-carotene branch (Fig. 1). Furthermore, *β-OHase* was down regulated during fruit development, decreasing further metabolism of β-carotene. Undoubtedly, these close associations cannot explain the color phenotype change, as all these gene expression patterns are maintained in the ‘green’ bulk as well (Fig. 6 and Additional file 1: Figure S4).

Since orange *vs* non-orange melon fruit flesh phenotype is determined by a single dominant gene (*CmOr)* and bulks of F_3_ families were analyzed, we can safely assume that the carotenoid biosynthesis capacity is similar in the two bulks. Fruit carotenoid level is the sum of synthesis, turnover rates and availability of storage capacity [16]. Since expression levels of biosynthetic and turnover genes are similar in green and orange melon fruit (Fig. 6), the accumulation of massive *β*-carotene in orange melon is likely due to the specific ability of *CmOr* in facilitating stable storage of carotenoids in chromoplasts, as was shown in *BoOr* cauliflower mutant and in transgenic potato overexpressing *BoOr* [55, 56]*.*

#### Sugar and carotenoid metabolism

We found a significant difference in mature fruit total solid soluble (TSS, Brix^0^) between the bulks. This suggested the existence of a direct or indirect metabolic link between fruit β-carotene and sugar accumulation or alternatively, a close genetic linkage between *CmOr* and a regulator of fruit sugar accumulation.

Carotenoid and soluble sugar accumulation levels were found to be correlated in various plant species and tissues. Recently a positive correlation between carotenoid and sucrose contents was demonstrated using melon recombinant inbred lines population derived from a cross between ‘*Dul*’ (sweet and orange) and PI414723 (non-sweet and pale orange) accession [57]. In the tomato fruit pericarp, lycopene accumulation was repressed by sucrose deficiency [58]. In citrus fruit epicarp, chloroplast to chromoplast transition (degreening) is initiated by elevation of soluble sugar levels and the process can be reversed (greening) by lowering sucrose concentration [59]. In transgenic maize, over-expression of carotenogenesis genes (*PSY1* and *CrtI*) influenced core metabolic processes in seed endosperm including a higher accumulation of sucrose [60].

In the present study, a comparison of green and orange fruit bulks of F_3_ families, derived from a cross between ‘*Dul*’ (sweet and orange flesh) and ‘*Tad*’ (very sweet and green flesh) revealed higher sucrose accumulation in green fruit segregants. The transcriptome data may provide an explanation to this result. Developing sweet melon fruits undergo a metabolic transition from a growing phase, during which stachyose and raffinose that are translocated to the young fruit are enzymatically processed and degraded, to a sugar accumulation phase, which is evident at the transcriptional level [33–35]. *Acid invertase* is down-regulated leading to a near cessation of sucrose degradation and *sucrose phosphate synthase* is up-regulated, boosting the synthesis of sucrose [33]. Together these changes make up part of the metabolic transition to sucrose accumulation [33]. We show here that genes involved in the transition to the fruit sucrose accumulation phase were differently expressed in the ‘green’ and ‘orange’ bulks during fruit development, most notably at 20 DAA (Fig. 7); *acid invertase* was down-regulated while *sucrose phosphate synthase* was up-regulated in the ‘green’ bulk compared to the ‘orange’ bulk.

Two alternative mechanisms could explain these transcriptional changes: a metabolic link, as shown recently in transgenic maize [60]; or a genetic linkage between *CmOr* and a gene (or genes) that regulates sucrose accumulation. MELO3C005363, annotated as *acid invertase,* is physically linked to *CmOr* (778,126 bp distant), and was differently expressed between the bulks (Fig. 7b). This physical proximity of *CmOr* and *acid invertase* is expected to result in segregants having the same parental alleles for both genes in most of the bulked fruits (Additional file 3: Table S3).

Final melon sucrose levels are primarily governed by the number of days passing from the decline in soluble acid invertase activity to fruit harvesting [34]. Down-regulation of acid invertase at 20 DAA in the ‘green’ bulk (Fig. 7) is most likely the cause for the higher sucrose levels in the green fruit, although we can’t exclude other unknown linked genes or a metabolic pleotropic effect of *CmOr*. To address the question of whether MELO3C005363 is the causal gene for the phenotype difference, F_4_ recombinant lines between *CmOr* and MELO3C005363 will be studied and analyzed in detail for sugar accumulation and metabolism.

## Conclusions

Comparative BSR-Seq analysis is a useful tool to reveal the transcriptomic impact of regulatory genes allelic variation. When utilizing the BSR-Seq approach, special care has to be taken regarding genes linked to the regulatory gene, as demonstrated in our study with acid invertase, which is linked to *CmOr* and possibly affected differences in fruit sugar content between the bulks. Nevertheless, our comparative approach revealed associations between *CmOr* allelic variation and cellular and metabolic processes during melon fruit ripening and generated a list of deferentially expressed genes that were affected by *CmOr* allelic variation. These genes and the processes that they are involved in are most probably part of the regulatory network governed by *CmOr* allelic variation. Sequence variations in some of these genes may be involved in the quantitative regulation of carotenoid accumulation in melon fruit and could be applied as new targets for breeding high carotenoid content melons.

## Methods

### Plant materials and bulk construction

A population segregating for fruit flesh color was constructed by crossing two previously characterized melon inbred lines: ‘Dulce’ (‘*Dul*’) and ‘Tam-Dew’ (‘*Tad*’). ‘*Dul*’ is an orange flesh, climacteric line belonging to the taxonomic group *reticulatus* and marketed as a Cantaloupe type. ‘*Tad*’ is a green flesh, non-climacteric line of the taxonomic group *Inodurus* and marketed as a Honey-Dew type. ‘*Dul*’ is homozygous dominant *CmOr,* while ‘*Tad*’ is homozygous recessive *Cmor.* This population was previously used to associate *CmOr* with the orange fruit phenotype, controlled dominantly by the *green-flesh* or *CmOr* gene [15]. As expected, the fruit flesh of the F_1_ offspring was always orange and the F_2_ fruit flesh color segregated to orange and green in the expected 3:1 ratio [15]. We self-pollinated F_2_ plants and defined each F_2_ plant offspring as an F_3_ family.

To generate bulk fruit material, 25 green and 25 orange fruit flesh homozygous F_3_ families (each family is originated from seeds of one F_2_ fruit) were chosen. DNA of first true leaf of 10 plantlets of each F_3_ family were pooled, amplified using the primers listed in Additional file 7: Table S1 and the amplicon was digested with *HinfI* enzyme, which cuts only the dominant allele (from orange flesh). The calculated chance to miss an orange segregating family was 1/3^10 or 1.69x10^−5^. Genotyping was validated by visualization of the mature flesh color of 12 plants of each selected F_3_ family. The calculated chance to miss an orange segregating family was 1/3^12 or 1.88x10^−6^. Thirty plants of each F_3_ family were grown in an open field during the summer of 2012 at the Newe Ya’ar research center in Northern Israel. Female flowers were marked on the day of anthesis. Three fruits from each family were picked at 10, 20, and 30 days after anthesis (DAA), as well as upon maturity (40–45 DAA). Twenty five fruits (one from each F_3_ family) were bulked to construct each biological repeat at each developmental stage. Three biological replicates were sampled. The large plant number grown in each F_3_ family allowed us to sample equal number of fruits for each color and developmental stage combination at every field sampling day, eliminating possible effects relating to time of maturation, circadian changes and environmental effects. Fruit flesh samples were immediately frozen in liquid nitrogen and kept in −80 °C until use.

### RNA extraction, library construction, and sequencing

Total RNA was extracted from bulked fruit mesocarp tissues following the protocol described by [61]. A total of 24 samples (2 genotypes x 4 developmental stages x 3 biological replications) bulked from 25 homozygous green or orange F_3_ families at different developmental stages were used for the strand-specific RNA-Seq library construction following the protocol previously described by [62]. Briefly, polyA mRNA was enriched by oligo(dT)^25^ Dynabeads from 5 ug of total RNA. The first strand cDNA was synthesized using SuperScript III reverse transcriptase (Invitrogen) and primer oligodT-VN (NEB). The second strand was formed using DNA polymerase. The synthesized cDNA was end-repaired and dA-Tailing. Barcode adapter was then added to each cDNA by T4 ligase, followed by purification and digestion with Uracil DNA glycosylase (NEB). The cDNA library was enriched by PCR using standard Illumina primers. AMPure XP beads were used to purify products after each step. The barcoded libraries (20 ng) were bulked and sequenced on a single lane of Illumina HiSeq 2000 sequencing system at the Cornell University core facility (http://www.brc.cornell.edu/brcinfo/).

### RNA-Seq data analysis

RNA-Seq reads were first aligned to the ribosomal RNA database [63] using Bowtie [64] allowing up to three mismatches and those that were aligned were discarded. The resulting reads were aligned to the melon genome [13] using TopHat [65] allowing one segment mismatch. The sequencing statistics and the correlation matrix are provided in Additional file 8: Table S7. Following alignments, raw counts for each melon gene were derived and normalized to reads per kilobase of exon model per million mapped reads)RPKM). The raw counts of melon genes were fed to edgeR [66] to identify differentially expressed genes (DEGs) between the ‘orange’ bulk and the ‘green’ bulk at each of the four developmental stages. Genes with adjusted *p*-value less than 0.01 and fold change greater than or equal to 2 were identified as DEGs. The DEGs were functionally classified using MapMan [23]. Changed metabolic pathways were identified using Plant MetGenMAP [32].

### SNPs identification

To minimize the artifacts of PCR amplification in SNPs identification, only one of the duplicated RNA-Seq reads in each library was used. To identify SNPs between the ‘green’ and the ‘orange’ bulks, RNA-Seq reads from the four stages of each bulk were first pooled and the pooled reads were aligned to the melon genome using BWA [67]. Only uniquely mapped reads (those having one single best hit to the melon genome) were kept. Following mapping, SNPs were identified based on the mpileup files generated by SAMtools [68]. The identified SNPs were supported by at least ten reads and had allele frequency of at least 0.9.

### Quantitative RT-PCR

To verify the RNA-Seq data, the cDNA was synthesized from the fruit RNA samples using Verso cDNA synthesis kit (Thermo Fisher Scientific). Quantitative RT-PCR (qRT-PCR) was conducted using the SYBR Green PCR master mix in an Applied Biosystems 7500 Real Time PCR System (Applied Biosystems, CA). PCR conditions were: denaturation at 95 °C for 20 s, 40 cycles of 95 °C for 3 s and 60 °C for 30s, 95 °C for 15 s, followed by 60 °C for 60s and gradual heating to 95 °C for melt curve construction. Relative expression levels were normalized with two housekeeping genes: melon *cyclophilin* and melon *ARP-1*. Primers were designed using primer 3 software and are listed in Additional file 7: Table S1. A melting curve analysis was performed for each reaction to confirm the amplification specificity. Real-time PCR was performed in triplicates. For ‘*Dul*’ and ‘*Tad*’ parental lines, we pooled fruit mesocarps of 6 fruits and homogenized them before RNA extraction. Cq values were determined by the ABI Prism 7000 SDS software and analyzed according to [69].

### Pigments analyses

Carotenoids were extracted in a mixture of hexane:acetone:ethanol (2:1:1, v/v/v) as described previously [70] and separated using a Waters 2695 HPLC apparatus equipped with a Waters 996 PDA detector (Milford, MA) [71]. Carotenoids were identified by their characteristic absorption spectra, distinctive retention time and comparison to authentic standards. Quantification was performed by integrating the peak areas with standard curves of authentic standards and the Waters millennium chromatography software. Lutein and two other unidentified carotenoids were relatively quantified at 450 nm by integrating their peaks areas and calculating its percentage from total integrated peaks areas.

Total chlorophylls were quantified according to [72]. Chlorophyll extracts were diluted 10 times in acetone and the absorbance of the samples was measured at 661.6 nm and 644.8 nm. Content of chlorophylls was calculated as follows:

$$ Ch{l}_a+Ch{l}_b\left(\mu g/ mL\  acetone\right) = \left(11.24\times {A}_{661.6}\hbox{--}\ 2.04\times {A}_{644.8}\right)+\left(20.13\times {A}_{644.8}\hbox{--}\ 4.19\times {A}_{661.6}\right) $$

### Fruit flesh color analysis

We used a Chroma-meter Konica-Minolta CR-400 as an unbiased method to define the visualized color phenotype of the sampled developing fruits. CIE color space L*, a* and b* values were obtained at three points of each fruit cross section. L* represents lightness (ranging from 0, black to 100, white), a* represents red (positive) to green (negative) axis, and b* represents yellow (positive) to blue (negative) axis. The colorimeter was calibrated on a white plate before each use.

### Sugar analysis

One gram of frozen mesocarp tissue of each of the 300 fruits comprising the bulks at 30 DAA and at the mature stage (25 families X 3 biologic repeats X 2 color phenotype X 2 developmental stages) was put in 80 % EtOH. Sugars were extracted and analyzed by HPLC as described [73].

### Availability of supporting data

The raw sequencing data has been deposited in NBCI SRA under the accession number SRP059243.

## Additional files

Additional file 1: Figure S1. Bulk phenotype during fruit development and parental line phenotype at the mature fruit stage. A. Average weight of fruits sampled for the BSAseq. Each point represents the mean of three repeats from each of the 25 F_3_ families that comprise each bulk. The green and orange dots and lines represent the green and orange flesh bulks respectively. The orange dots and line represent the orange flesh bulk. X axis is DAA. No significant fruit weight differences were detected. B. Mature fruit weight of the parental lines. Each column represents the average of 6 fruits. Error bars are standard error of the mean. C. Histograms show proportion of different weight groups (Kg) in the entire population (gray columns) and in the sampled orange and green fruit (orange and green columns respectively). The orange and green subpopulations display normal distribution around the mean according to Goodness of Fit test. (Jump-8 software). C. Chroma-meter (Minolta Sensing Inc, Minolta Chroma Meter Model CR-400, Osaka, Japan) measurements of the developing fruits described in A. Each cut-open fruit was measured at three points of the mesocarp center. The Y axes colored bars illustrate the color range of a*, b* and l* values (see material and methods). D. Chroma-meter measurements of mature fruits of the parental inbred lines, F_1_ and the bulks. Dulce, Tam-Dew and F_1_ columns represent the average of six fruits. The bulks mature fruit are the same measurements as in C. E. Total soluble solids (TSS) values in the fruit flesh measured by optical refractometer at three developmental stages. The TSS values of ‘green’ and ‘orange’ bulks at the mature stage are statistically different (*P* < 0.05). F. One-way analysis of TSS variance by bulk colors. Tukey-Kremer HSD test was measure with Jump-8 software (SAS Institute, Inc., NC). G. Mature fruit TSS of the inbred parental lines. Differences are statistically significant (*P* < 0.05). Fruits are as in B. **Figure S2.** intermediate metabolites. Two dimensional HPLC chromatograms of orange and green mature bulks fruit (for more information see Fig. 3e legend). Measuring absorbances at 290 nm and at 400 nm enabled visualizing the peaks of phytoene and ζ-carotene respectively, in the orange but not in the ‘green’ bulk. **Figure S3.** Heat map presentation and correlation analysis of qRT-PCR verification of DEGs in bulks and in parental lines: A. Each row in the map represents one DEG (from left to right): ID names (https://melonomicas.net); RNA-seq based log ratio of digital expression (colored scale bar on top of the map) during fruit development; log ratio of relative expression based on qRT-PCR analysis of bulks and parental lines cDNA at one developmental stage outlined in the RNA-seq column section. B. Correlation analysis between digital and relative genes expression: X axis is qRT-PCR based log ratio of relative expression. Y axis is RNA-seq based log ratio of digital expression. Correlation coefficient (r) was 0.943 showing high and statistically significant correlation (*P* < 0.00001). **Figure S4.** ABA-2 gene expression in ‘green’ and ‘orange’ bulks during fruit development. Each bar is the average RPKM of three biological repeats. Each biological repeat includes 25 bulked fruits, one from each F3 family, at four developmental stages presented on the x axis. Error bars represent the standard error of the mean. **Figure S5.** MEP pathway genes expression at different fruit development stages. Each bar is the average RPKM of three biological repeats. Each biological repeat includes 25 bulked fruits, one from each F_3_ family, at four developmental stages presented on the x axis. Error bars represent the standard error of the mean. Genes are: deoxy-d-xylulose 5-phosphate (DXP) synthase (*DXS*), DXP reductase (*DXR*) and Geranylgeranyl pyrophosphate synthase (*GGPPS*). ID names from (https://melonomicas.net). (DOCX 1141 kb) Additional file 2:
**Table S1**. List of primers. (XLSX 12 kb) Additional file 3: Table S2. SNP differentiating between bulks. *: same base as reference genome; SNP type: M, mismatch; I, insertion; D, deletion; AA change: SNP causes amino acid changes; for example, R108H means that SNP at CDS position 108 causes amino acid changes from R to H (“R” in green bulk to “H” in orange bulk). (XLSX 16 kb) Additional file 4: Table S3. List of genes included in the significant SNP area. (XLSX 19 kb) Additional file 5: Table S4. List of 79, 805, 37 and 122 genes that were differentially expressed at 10, 20, 30 DAA, and the mature stage, respectively. (XLSX 162 kb) Additional file 6: Table S5. List of genes exhibited significant up or down regulation in two fruit developmental stages. (XLSX 10 kb) Additional file 7: Table S6. DEGs at different fruit developmental stages categorized into functional groups using MapMan. (XLSX 4378 kb) Additional file 8:
**Table S7**. RNA-sequencing statsitics. (XLSX 16 kb)

## Abbreviations

‘*Dul*’: Dulce
‘*Tad’*: Tam-Dew
ABA: Abscisic acid
BSA: Bulk segregant analysis
BSR-Seq: Bulk segregant RNA-Seq
CRTISO: Carotenoid isomerase
DAA: Days after anthesis
DEG: Differentially expressed gene
ɛ-LCY: Lycopene ɛ-cyclase
FW: Fresh weight
GGPP: Geranylgeranyl diphosphate
GGR: Geranylgeranyl reductase
MEP: C-methyl-D-erythritol 4-phosphate
PDS: Phytoene desaturase
PSY: Phytoene synthase
RPKM: Reads per kilobase, per million sequenced reads
ZDS: ζ-carotene desaturase
Z-ISO: ζ-carotene isomerase
β-LCY: Lycopene β-cyclase
β-OHase: β-carotene hydroxylase
