# Supplementary figures and images for: A bulk segregant transcriptome analysis reveals metabolic and cellular processes associated with Orange allelic variation and fruit β-carotene accumulation in melon fruit
Source: BMC Plant Biol. 2015 Nov 9;15:274. doi: 10.1186/s12870-015-0661-8 (PMC4640158; doi:10.1186/s12870-015-0661-8)

**Figure S1:**

**
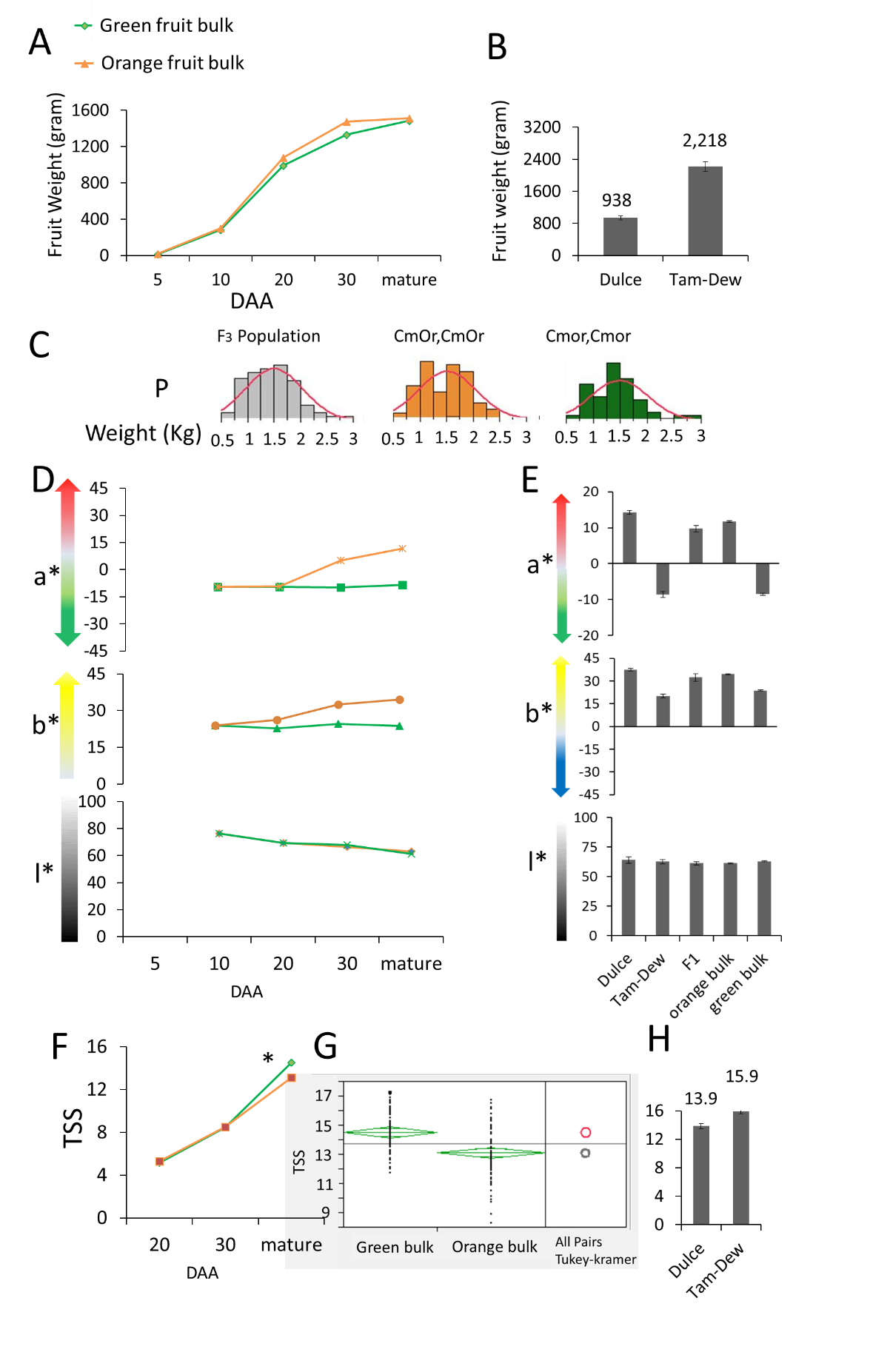
**

**Figure S2:**

**
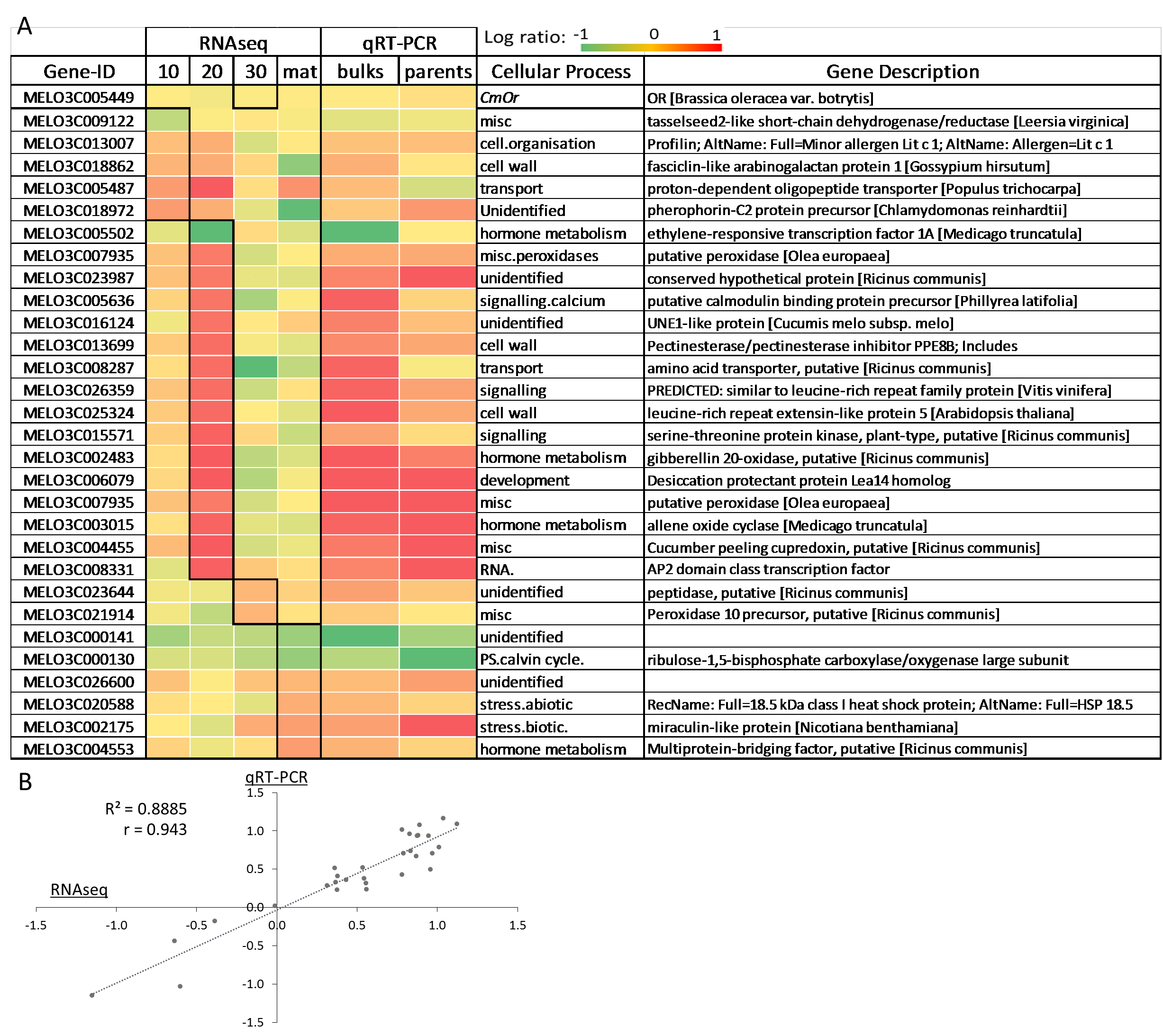
Figure S3:**

**Figure S4:**


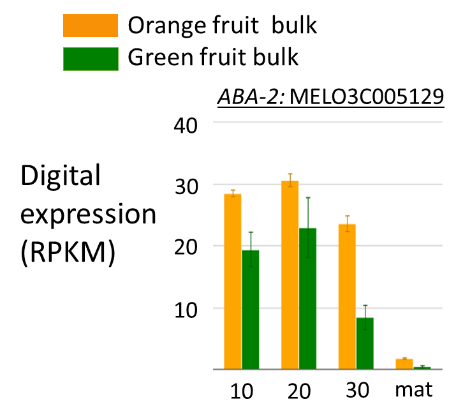


**Figure S5:**


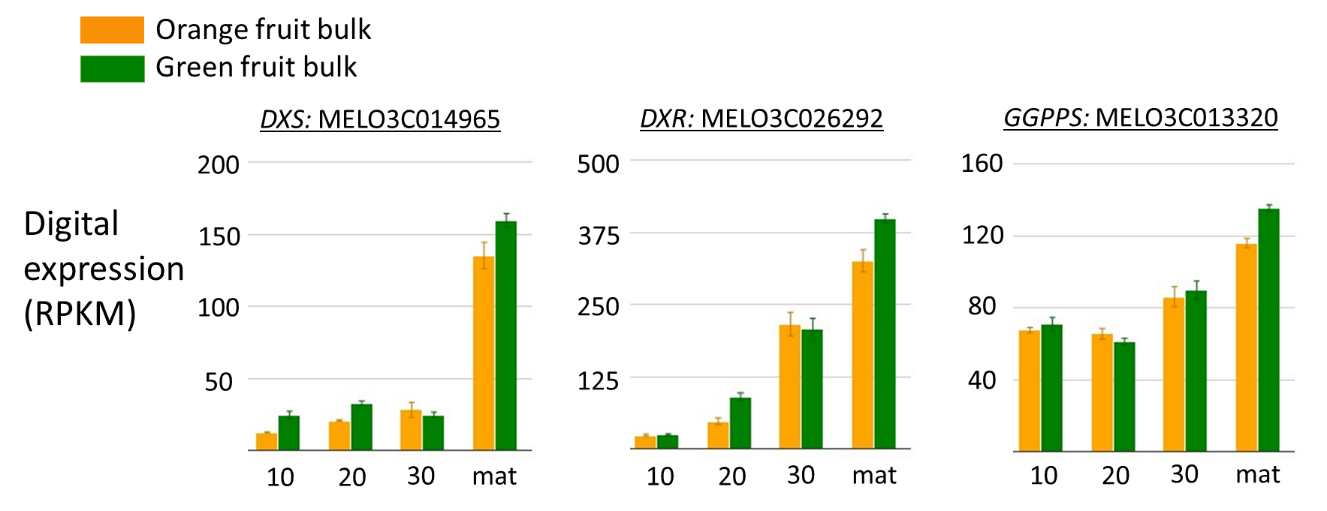

Supplement: Additional file 1: Figure S1. — Bulk phenotype during fruit development and parental line phenotype at the mature fruit stage. A. Average weight of fruits sampled for the BSAseq. Each point represents the mean of three repeats from each of the 25 F3 families that comprise each bulk. The green and orange dots and lines represent the green and orange flesh bulks respectively. The orange dots and line represent the orange flesh bulk. X axis is DAA. No significant fruit weight differences were detected. B. Mature fruit weight of the parental lines. Each column represents the average of 6 fruits. Error bars are standard error of the mean. C. Histograms show proportion of different weight groups (Kg) in the entire population (gray columns) and in the sampled orange and green fruit (orange and green columns respectively). The orange and green subpopulations display normal distribution around the mean according to Goodness of Fit test. (Jump-8 software). C. Chroma-meter (Minolta Sensing Inc, Minolta Chroma Meter Model CR-400, Osaka, Japan) measurements of the developing fruits described in A. Each cut-open fruit was measured at three points of the mesocarp center. The Y axes colored bars illustrate the color range of a*, b* and l* values (see material and methods). D. Chroma-meter measurements of mature fruits of the parental inbred lines, F1 and the bulks. Dulce, Tam-Dew and F1 columns represent the average of six fruits. The bulks mature fruit are the same measurements as in C. E. Total soluble solids (TSS) values in the fruit flesh measured by optical refractometer at three developmental stages. The TSS values of ‘green’ and ‘orange’ bulks at the mature stage are statistically different (P < 0.05). F. One-way analysis of TSS variance by bulk colors. Tukey-Kremer HSD test was measure with Jump-8 software (SAS Institute, Inc., NC). G. Mature fruit TSS of the inbred parental lines. Differences are statistically significant (P < 0.05). Fruits are as in B. Figure S2. intermediate metabolites. Two dimensiona [file 12870_2015_661_MOESM1_ESM.docx]
